# Terbutaline versus salbutamol for suppression of preterm labor: a randomized clinical trial

**DOI:** 10.4103/0256-4947.67079

**Published:** 2010

**Authors:** Shahdokht Motazedian, Fariborz Ghaffarpasand, Khatereh Mojtahedi, Nasrin Asadi

**Affiliations:** a Department of Obstetrics and Gynecology, Shiraz University of Medical Sciences, Shiraz, Iran; b Student Research Committee, Fasa University of Medical Sciences, Fasa, Iran

## Abstract

**BACKGROUND AND OBJECTIVE:**

Preterm labor (PTL) is a common medical problem during pregnancies and is associated with neonatal mortality and morbidity. Beta-adrenergic agonists are among the most commonly used tocolytic agents. The aim of this study was to compare the effectiveness, safety and adverse effects of terbutaline with those of salbutamol in the prolongation of pregnancy beyond 48 hours and until 37 weeks of gestation.

**PATIENTS AND METHODS:**

Two hundred women with PTL were randomly assigned to receive subcutaneous terbutaline (250 μg) or intravenous salbutamol (0.1 mg) followed by oral terbutaline (20 mg/d) or oral salbutamol (24 mg/d) as maintenance. The efficacy, side effects and complications after 48 hours and until 37 weeks of gestation were analyzed and compared.

**RESULTS:**

There was no significant difference between the two groups in success rate within 48 hours (*P*=.091). Gestational age at delivery (*P*=.031) and the number of days for which the gestation was prolonged (*P*=.024) were significantly higher in those receiving terbutaline. Adverse effects, including tachycardia (*P*=.007) and anxiety (*P*=.006), were experienced more in the salbutamol group. Birth weight was significantly lower in the salbutamol group (*P*=.001).

**CONCLUSION:**

Terbutaline provided more effective tocolysis with fewer adverse effects and a better neonatal outcome. However, terbutaline and salbutamol are equally effective in the first 48 hours.

Preterm labor (PTL) occurs in 5% to 10% of all pregnancies worldwide^1^ and is defined as labor occurring prior to 37 weeks of gestation. PTL is also the most common cause of neonatal mortality and morbidity, after congenital malformations. More than 10% of infants born before 28 weeks of gestation are severely handicapped. It has also been shown that severe neonatal morbidity, such as respiratory distress syndrome (RDS) and intraventricular hemorrhage (IVH), decreases with increasing gestational age.^2^ Newborns of less that 31-32 weeks gestation suffer more from neurological and sensorial deficiencies.^3^,^4^

It is essential to suppress uterine contractions in patients with PTL (except in those with intrauterine infections and fetal distress) so as to postpone pregnancy until the fetus becomes mature enough. Until now many medications or interventions have been introduced for prolongation of pregnancies complicated with PTL. However, none have been found to be completely effective, and the choice of drug is often limited by adverse effects.^5^,^6^ Beta-adrenergic agonists are among the most commonly used tocolytic agents. It has been shown that these agents postpone the delivery for 24, 48 hours and even 7 days. However, such a delay has not been associated with a significant reduction in either perinatal mortality or morbidity.^7^,^8^

Postponement of pregnancy for at least 48 hours allows the corticosteroids to reach their maximum effect for reducing RDS and its sequelae, as well as IVH.^9^ Previous studies have shown that both subcutaneous terbutaline^10^–^12^ and intravenous salbutamol^13^–^15^ are effective tocolytic agents for patients with PTL. However, no study has compared the effects of these two agents until now. Accordingly, this study aimed to compare the effectiveness, safety and adverse effects of terbutaline with those of salbutamol in the prolongation of pregnancy until and beyond 48 hours.

## PATIENTS AND METHODS

This randomized clinical trial was performed at the labor ward of Zeinabieh Hospital, a tertiary care center affiliated with Shiraz University of Medical Sciences. We screened 287 patients with PTL, ranging in age from 17 to 32 years, between April 2009 and September 2009. The respective approvals of the review board and the ethics committee of the Shiraz University of Medical Sciences were obtained before proceeding with the study. The study protocol and its benefits and complications were explained to all participants, and all recruited patients completed and signed the ‘informed consent’ form.

All women who presented to the labor ward between 20 and 37 weeks of pregnancy (determined by the date of the last menstrual period when known or by early ultrasound) were assessed for enrollment into the study. Preterm labor was defined as the persistence of at least two symptomatic uterine contractions within a 10-minute period during the 60 minutes after admission and despite bed rest in the presence of cervical dilation between 0 and 3 cm for primigravida and between 1 and 3 cm for multigravida, with cervical effacement less than 50%. Women with cervix dilatation greater than 5 cm, polyhydramnios [amniotic fluid index (AFI) greater than 20 cm], oligohydramnios (AFI less than 10 cm), macrosomia (estimated fetal weight of 4 kg or more), suspected intrauterine infection (a temperature greater than 37.5°C) or growth restriction, major antepartum hemorrhage, rupture of membranes, major maternal medical disorders, diabetes mellitus, presence of a contraindication for vaginal delivery (e.g., major placenta previa), one previous cesarean delivery, multiple pregnancy, hypertension in pregnancy, blood pressure less than 90/50 mm Hg, previous history of abruption placenta, signs of fetal distress, lethal fetal anomaly and contraindication for the use of beta-sympathomimetic drugs and previous treatment with tocolytics in the present gestation were excluded from the trial.

After performing biochemical and hematological blood tests and an electrocardiogram, women were randomly assigned to two study groups using a computerized random number generator in a sequence of sealed, numbered opaque envelopes. There was a 1:1 randomization ratio. Women in the first group received subcutaneous terbutaline (Tehran Chimi Drug, Tehran, Iran) in a dosage of 250 μg (loading dose) followed by the same dose every 45 minutes if the uterine contractions persisted. Women in the second group received intravenous salbutamol (Tehran Chimi Drug, Tehran, Iran) in a dosage of 0.1 mg (bolus dose) followed by same boluses every 5 minutes until discontinuation of contractions. After stoppage of the uterine contractions, women in the first group received 20 mg/day oral terbutaline in 4 divided doses while women in the second group received 24 mg/day oral salbutamol in 4 divided doses as maintenance. All study personnel and participants were blinded to treatment assignment for the duration of the study. Only the study statisticians and the data monitoring committee saw unblinded data, but none had any contact with study participants.

Fetal heart rate and uterine contractions, as well as maternal blood pressure and pulse rate, were assessed every 12 hours. Prolongation of pregnancy for a period of 48 hours was the primary outcome in this study. The intervention was considered a failure in participants who delivered during the period, or still had contractions after 48 hours; and it was considered a success in participants whose contractions stopped for the full 48 hours. Patients whose contractions discontinued after 48 hours were discharged from the hospital with the maintenance therapy. If uterine contractions reappeared, relapse was diagnosed and treatment was repeated as indicated above. Medication was continued until 37 weeks of gestation. The participants were instructed to report in a daily diary the onset of any adverse experiences, specifying the severity, duration and a possible cause-effect relationship with drug administration. Neonatal weight, Apgar score, umbilical arterial and venous pH values and the presence of hyperbilirubinemia were determined after delivery. Neonatal complications such as hemorrhage or infections were also recorded. The secondary outcome of the study was the length of pregnancy and the side effects of each medication. Prolongation of gestation from admission to hospital to delivery and the number of preterm deliveries before 37 weeks (259 days) of gestation were also assessed.

Seventy-five patients were required in each group for the study to have 90% power to detect 10% difference between two groups regarding success rate (*P*=.05, 2-sided). To compensate for possible non-evaluable data, we enrolled 100 participants in each group. The statistical software package SPSS for Windows, version 15.0 (SPSS, Chicago, IL, USA), was used for data analysis. Paired t tests were used to compare results within groups; independent t tests were used to compare results between the groups; χ^2^ tests were used to compare proportions. Multivariate logistic regression analyses were carried out to control for the potentially confounding effect of important baseline factors. Data were reported as mean (standard deviation) for 95% confidence intervals. A 2-sided *P* value <.05 was considered statistically significant.

## RESULTS

**Figure 1:**
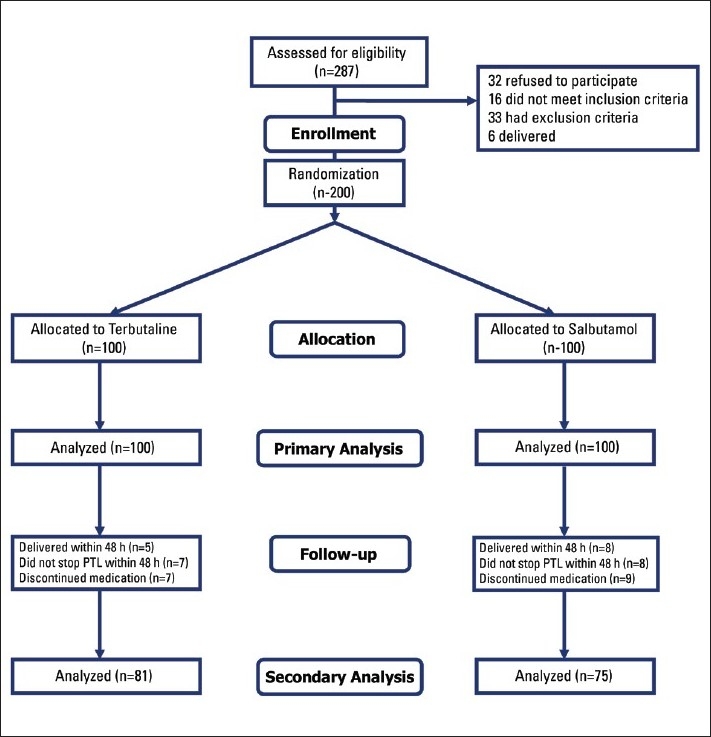
Flowchart of the study.

A total of 287 women were assessed for eligibility; 200 were eligible and were randomized into two study groups. All finished the study (Figure 1). None of the demographic and baseline parameters differed significantly between the two groups (Table 1). A 48-hour prolongation of pregnancy was achieved in 87 (87%) patients receiving terbutaline and 84 (84%) patients receiving salbutamol, which was not statistically significant (*P*=.091) (Table 2). There was also no difference in the delivery rate within 48 hours (*P*=.072). The patients in the salbutamol group experienced more complications (*P*<.001), including tachycardia (*P*=.007), palpitation (*P*=.002), anxiety (*P*=.006) and chills (*P*<.001).

**Table 1:** Baseline characteristics of the two study groups.

Characteristic	Terbutaline group (n=100)	Salbutamol group (n=1oo)	*p*^a^
Maternal age (years)	22.3 (8.83)	23.2 (5.75)	.098
Parity	1.31 (0.78)	1.46(1.21)	.083
Gestational age (weeks)	30.4 (3.16)	31.1 (5.43)	.086
Cervical dilatation (cm)	1.56 (0.47)	1.48 (0.51)	.224
Cervical effacement (%)	40.6 (32.1)	39.8 (29.3)	.064
Contractions interval (minutes)	4.63 (0.62)	4.23(1.13)	.155
Contractions duration (seconds)	35.6 (4.48)	34.8 (5.12)	.092

Values are mean (standard deviation

a Independent sample *t* test

**Table 2:** Outcome in women with preterm labor after 48 hours.

Characteristic	Terbutaline group (n=100)	Salbutamol group (n=100)	*p*
Prolongation within 48 hours (n,%)	87 (87)	84 (84)	.091^a^
Delivery within 48 hours (n, %)	5 (5)	8 (8)	.072^a^
Duration of admission (days)	3.2 (1.4)	4.0 (1.4)	.096^b^
Side effects (n, %)	41 (41)	76 (76)	<.001^a^
Tachycardia (n, %)	20 (20)	42 (42)	.007^a^
Dyspnea (n, %)	5 (5)	12 (12)	.066^a^
Nausea (n, %)	19 (19)	29 (29)	.058^a^
Palpitation (n, %)	9 (9)	31 (31)	.002^a^
Anxiety (n, %)	15 (15)	31 (31)	.006^a^
Chills (n, %)	28 (28)	58 (58)	<.001^a^
Edema (n, %)	3 (3)	4 (4)	.161^a^

Values are mean (standard deviation) or number (percent)

a Chi-square test

b lndependent sample *t* test

Seven women in the terbutaline group and 9 in the salbutamol group discontinued maintenance medication due to complications (P=.048); thus, 81 and 75 women, respectively, were available for secondary analysis (Figure 1). Table 3 shows the secondary outcome and the pregnancy outcome in the two study groups. Gestational age at delivery was significantly higher in those receiving terbutaline (*P*=.031). Women receiving salbutamol experienced more relapses compared to those who received terbutaline (*P*=.001). Birth weight was significantly lower in those who received salbutamol as a tocolytic agent (*P*=.001). In the same way, the prevalence of low birth weight (<2500 g) was higher in the salbutamol group (*P*=.004). There was no significant difference between groups in Apgar score (*P*>.05) and perinatal mortality (*P*=.606). We performed a multivariate logistic regression analysis incorporating maternal age, gestational age, cervical dilatation, cervical effacement, contraction intervals, contraction duration and allocated treatment as independent covariables in the analysis, with gestational age at delivery and length of pregnancy (days) separately as dependent outcomes. After controlling for independent factors in both models for gestational age at delivery and length of pregnancy, terbutaline tocolysis remained significantly associated with increased gestational age at delivery and increased length of pregnancy.

**Table 3:** Outcome in women with preterm labor after 37 weeks of gestation.

Characteristic	Terbutaline group (n=81)	Salbutamol group (n=75)	*p*
Gestational age at delivery (weeks)	38.9 (1.5)	37.1 (1.8)	.031^a^
Prolongation of gestation (days)	59.5 (12.6)	42.3 (11.3)	.024^a^
Number of PTL relapses (%)	26 (32.1%)	49 (65.3%)	.001^b^
Discontinuing due to complications (%)	7 (8.6%)	9 (12%)	.048^b^
Cesarean delivery (%)	11 (13.6%)	10 (13.3%)	.784^b^
**Pregnancy outcome**			
Birth weight (g)	3281 (382.23)	3011 (508.7)	.001^a^
Low birth weight (%)	6 (7.4%)	10 (13.3%)	.004^b^
Apgar score in 1 min	8.39 (0.8)	8.38 (0.8)	.266^a^
Apgar score in 5 min	9.29 (0.3)	9.14 (0.2)	.097^a^
Live births (%)	79 (97.5%)	74 (98.6%)	.171^b^
Perinatal mortality (%)	2 (2.5%)	1 (1.4%)	.606^b^
Respiratory distress syndrome (%)	1 (1.2%)	3 (4%)	.275^b^
Neonatal sepsis (%)	1 (1.2%)	0 (0%)	.334^b^

Values are mean (standard deviation) or number (percent).

a Independent sample *t* test

b Chi-square test

## DISCUSSION

In this study, we compared the efficacy, safety and adverse effects of terbutaline with those of salbutamol in suppression of PTL and maintaining a normal pregnancy until term delivery. To the best of our knowledge, this is the first study comparing terbutaline and salbutamol for prolongation of pregnancy in patients with PTL. We found that there was no difference between these two beta-adrenergic agonists in the success rate and delivery rate within the first 48 hours. However, length of pregnancy was significantly higher in those who received terbutaline. Adverse drug effects were significantly greater in the salbutamol group. These findings show that terbutaline is more effective in suppressing PTL while prescribed as maintenance for the long term. It is also accompanied by fewer side effects and complications. Salbutamol was associated with an increased prevalence of low birth weight. However, there was no statistically significant difference in the Apgar scores or other complications. Terbutaline was safer and better tolerated and had a better pregnancy outcome.

Our findings are consistent with previous studies that have shown that terbutaline^10^–^12^ and salbutamol^13^–^15^ are effective tocolytic agents accompanied by side effects. These studies show that beta-adrenergic agonists are more effective compared to calcium channel blockers;^10^–^12^,^14^ however, their significant side effects have limited use in practice. On the other hand, these agents do not decrease the neonatal mortality and morbidity associated with PTL. Thus, the popularity of these agents as first-line tocolytic agents has decreased.^16^,^17^ Nevertheless, these agents are recommended as first-line tocolytic agents because of their efficacy.^12^,^14^

Phupong et al^13^ studied the effect of oral salbutamol (32 mg/d) on prolongation of pregnancy in those with PTL. Of the 132 pregnancies, 81.1% were prolonged for more than 24 hours; 59.8%, for more than 2 days; 32.6%, for more than 1 week; and 8.3%, for more than 4 weeks. Tachycardia was the most common side effect, which was experienced by 85.6% of the individuals. Neonatal complications occurred in 28% of the babies, while respiratory distress syndrome occurred in 22.7% of the babies. The pregnancy outcome was significantly better in the group that had a prolongation time of at least 48 hours. In this study, we used both subcutaneous and intravenous boluses of terbutaline and salbutamol, respectively, for suppression of PTL within 48 hours and oral maintenance (20 mg/d for terbutaline and 25 mg/d for salbutamol until 37 weeks of gestation) for prevention of PTL. Tachycardia and chills were the most common side effects observed in those who received salbutamol. Our findings are consistent with those in the study by Phupong et al,^13^ which found that oral salbutamol is associated with severe side effects and poor pregnancy outcome.

Mawaldi et al^10^ compared the effects of subcutaneous terbutaline with those of oral nifedipine for prolongation of gestation. Terbutaline and nifedipine appeared to be equally effective in their tocolytic action. However, nifedipine did have the advantage of ease of administration. It also had significantly lesser effect on the fetal heart rate and was accompanied by lesser side effects. It was also shown by Vani et al^15^ that adjusted-dose intravenous salbutamol tocolysis prior to external cephalic version increases its success rate and reduces the cesarean delivery rate. Oral terbutaline was more effective in preventing PTL until 37 weeks of gestation, as demonstrated by a lesser number of relapses during this period.

The oral form of terbutaline and salbutamol was used as maintenance in this study because it does not require intensive medical nursing care and observations and is not associated with the discomfort of an intravenous line. The dosage used in this study is equivalent to that commonly used, intravenous salbutamol infusion dose of 6-30 μg/min, to inhibit preterm labor.^18^ Beta-adrenergic agonists increase the blood glucose levels by promoting glucagon release, gluconeogenesis and insulin resistance.^19^ Although none of our patients developed increased blood glucose, it must be kept in mind that women with diabetes were excluded from the study. This effect may therefore be of concern in diabetic women.

In conclusion, the results of this study demonstrate that terbutaline and salbutamol appeared to be equally effective tocolytic agents within 48 hours. However, oral terbutaline was more effective in preventing PTL until 37 weeks of gestation and was accompanied by fewer side effects and a better neonatal outcome. These findings make terbutaline the drug of choice for prolongation of pregnancy in those women with PTL.
